# Measuring surgical safety during minimally invasive surgical procedures: a validation study

**DOI:** 10.1007/s00464-018-6021-7

**Published:** 2018-01-19

**Authors:** Mathijs D. Blikkendaal, Sara R. C. Driessen, Sharon P. Rodrigues, Johann P. T. Rhemrev, Maddy J. G. H. Smeets, Jenny Dankelman, John J. van den Dobbelsteen, Frank Willem Jansen

**Affiliations:** 1 Department of Gynecology, Leiden University Medical Center, P.O. Box 9600, 2300 RC Leiden, The Netherlands; 2 Department of Gynecology, Haaglanden Medical Center, P.O. Box 96900, 2509 JH The Hague, The Netherlands; 3 Department of BioMechanical Engineering, Delft University of Technology, Mekelweg 2, 2628 CD Delft, The Netherlands

**Keywords:** Interobserver reliability, Minimally invasive surgery, Video observation, Surgical safety

## Abstract

**Background:**

During the implementation of new interventions (i.e., surgical devices and technologies) in the operating room, surgical safety might be compromised. Current safety measures are insufficient in detecting safety hazards during this process. The aim of the study was to observe whether surgical teams are capable of measuring surgical safety, especially with regard to the introduction of new interventions.

**Methods:**

A Surgical Safety Questionnaire was developed that had to be filled out directly postoperative by three surgical team members. A potential safety concern was defined as at least one answer between (strongly) disagree and indifferent. The validity of the questionnaire was assessed by comparison with the results from video analysis. Two different observers annotated the presence and effect of surgical flow disturbances during 40 laparoscopic hysterectomies performed between November 2010 and April 2012.

**Results:**

The surgeon reported a potential safety concern in 16% (85/520 questions). With respect to the scrub nurse and anesthesiologist, this was both 9% (46/520). With respect to the preparation, functioning, and ease of use of the devices in 37.5–47.5% (15–19/40 procedures) a potential safety concern was reported by one or more team members. During procedures after which a potential safety concern was reported, surgical flow disturbances lasted a higher percentage of the procedure duration [9.3 ± 6.2 vs. 2.9 ± 3.7% (mean ± SD), *p* < .001]. After procedures during which a new instrument or device was used, more potential safety concerns were reported (51.2 vs. 23.1%, *p* < .001).

**Conclusions:**

Potential safety concerns were especially reported during procedures in which a relatively high percentage of the duration consisted of surgical flow disturbances and during procedures in which a new instrument or device was used. The Surgical Safety Questionnaire can act as a validated tool to evaluate and maintain surgical safety during minimally invasive procedures, especially during the introduction of a new intervention.

**Electronic supplementary material:**

The online version of this article (10.1007/s00464-018-6021-7) contains supplementary material, which is available to authorized users.

In the ongoing search for optimal patient outcomes, surgical procedures are continuously evolving [1]. As a result, maintaining the high level of patient safety has become a great challenge [2]. Implementing new techniques and/or technologies causes changes in standardized surgical procedures to which every surgical team member has to adapt [3, 4]. Monitoring surgical safety in the operating room (OR) is one of the most important issues to guarantee optimal surgical outcome. However, real-time monitoring of the surgical safety during a procedure is difficult. The question is: what and how should we monitor and who should do it?

Previous studies describing patient safety during minimally invasive surgery (MIS) have defined certain domains that are ‘at risk’ [5–8]. In daily practice the identification of these safety issues is often limited to observers that were physically present in the OR and retrospective interpretation of the obtained data [6, 9, 10]. Adequate interpretation is difficult and requires correct differentiation of *errors* (undesired actions) from *events* (consequence of undesired actions) [5]. Currently, patient safety indicators are frequently based on the occurrence of adverse events [11]. However, in general, intraoperative adverse events rarely occur. In theory, for an adverse event to occur several errors have to line up and slip through the holes of existing safety barriers [12]. Usually most errors that precede a potential adverse event are timely recognized and dealt with. However, these near-misses disturb the surgical flow to a greater or lesser extent and therefore interfere with surgical safety [3–5, 10, 13–16].

In daily practice, there is no external observer present during a procedure. The only ‘real-time monitoring’ of patient safety is done by the surgeon and/or the entire surgical team itself. However, from a psychological perspective it is known that an individuals’ situational awareness is impaired when occupied with a (difficult) task [17]. Regarding this phenomenon, implementing new surgical devices and technologies in the OR puts more pressure on the responsibility of the surgeon to maintain surgical safety during the whole procedure [1, 15]. The only measures to enhance safety throughout a procedure that currently are—or at least should be—used, are the preoperative team briefings, the postoperative debriefings and, to a lesser extent, some preoperative checklists. In general, these safety instruments have proven to diminish preventable errors during the procedure and to safeguard open communication [18–21]. However, since these tools do not incorporate items to evaluate new surgical techniques or technologies, they are insufficient in detecting safety hazards during their introduction.

Therefore, the aim of this study was to observe whether surgical teams are capable of measuring surgical safety, especially with regard to the introduction of new techniques and technologies during a series of MIS procedures. A questionnaire that had to be filled out directly postoperative was developed to measure surgical safety. Next, the validity of the questionnaire was assessed by comparison with the results from independent video analysis of these procedures.

## Materials and methods

In a university-affiliated teaching hospital (Haaglanden Medical Center, The Hague), a prospective registration study was set-up to record and analyze surgical flow disturbances. During a consecutive series of laparoscopic hysterectomies (LH), a questionnaire was filled out in the OR by the surgical team members. The surgical flow disturbances were scored by an independent observer. To minimize the interference of the study on its own results (the ‘Hawthorne effect’), this observation was based on video registration of the procedures. Outcome measures were the number, types, effect, and duration of surgical flow disturbances per procedure.

The LH was chosen as procedure of interest, because it is an advanced laparoscopic procedure performed by a dedicated operating team and requiring a wide array of endoscopic instruments and equipment. The study started in November 2010 and all consecutive LHs that were performed in a conventional (cart-based) OR were registered until the start of the construction of the new integrated OR (Karl Storz OR1™ integrated OR system, September 2011). After construction of the integrated OR (October 2011), the same amount of eligible procedures was registered in this setting. Similarly, the occasional introduction of new devices in both the conventional and integrated OR was registered. In this manner, not only the transition to the integrated OR, but also the introduction of new devices was analyzed. All procedures were performed by either of the two gynecologists with more than 10 years of experience in advanced gynecologic laparoscopy and were assisted by one gynecologist who conducted a fellowship in MIS; a group of five alternated in the position of either circulating or scrub nurse.

The study was approved by the Executive Board of the Haaglanden Medical Center. Prior to the start of the study, all OR personnel were collectively informed about the study. From each patient, informed consent was obtained. This design was adapted from another study [3].

### Development of Surgical Safety Questionnaire

Patient safety risk factors that have been described by Rodrigues et al., were summarized in a questionnaire consisting of 13 questions (i.e., time-out/sign-out, preparation and functioning of devices and instruments, functioning of the surgical team, distracting stimuli, and interference of the study on the procedure) [6]. Directly after each procedure the (assisting-)surgeon, scrub nurse, and anesthetist(-assistant) filled out this short questionnaire. Answers were given on a 5-point Likert scale, ranging from (strongly) disagree to (strongly) agree. A potential safety concern was defined as an answer between (strongly) disagree and indifferent by at least one member of the surgical team. Additionally, several questions regarding experience (with the procedure, laparoscopy in general, and the used instruments/devices) and the procedure (adhesions, adverse events) were stated (see Online Appendix).

### Video analysis

The input from three video signals (endoscopic image and two dome cameras) and four audio signals (MPEG Recorder 2.1) was synchronously recorded during all procedures. The recordings were started just before the time-out procedure and stopped after suturing all port-sites. The procedure was excluded from analysis in case of technical problems related to the recording equipment. Two residents in Obstetrics and Gynecology (M.D.B. and S.R.C.D.) analyzed the presence and effect of predefined surgical flow disturbances. These surgical flow disturbances were defined as stimuli distracting one or more members of the surgical team (Table 1). To assess the severity, the effect of the surgical flow disturbance on the surgical team members was graded according to a seven-point scale. This scale ranges from 1 as a potentially distracting stimulus to 7 when the sterile team’s work is completely interrupted (modified by Persoon et al. originally described by Healey et al.) (Table 2) [9, 22].

**Table 1:** Observed types of surgical flow disturbances

Equipment-/instrument-related
Set-up device/connection
Intraoperative repositioning
Malfunctioning
Not present
Sterility
Other/unclear
Environmental
Pager/telephone
Door washing room
Radio use
Personnel-related
Communication failure
Irrelevant conversation
Procedure-related
Extra coagulation bleeding-site
Unexpected adhesions
Limited vision (condensation/smoke)
Adverse event
Conversion to laparotomy

**Table 2:** Effect of observed surgical flow disturbances (according to Persoon et al. [9])

1	Events with the potential to distract the sterile team
2	Sterile team member momentarily distracted: possible involvement of a single sterile member in an event not related to the primary task, e.g., a short head turn in response to a visual or auditory stimulus
3	Sterile team member engages in distraction: similar distraction in 2, but the sterile member engages with the source of distraction by verbally responding while maintaining primary task activity (multitasking)
4	Sterile team member’s primary task interrupted: a single team member ceases his/her current tasks to engage entirely in the distracting stimulus
5	Sterile team momentarily distracted: two or more sterile team members respond to a stimulus with a short head turn, no verbal response
6	Sterile team engage in secondary tasks: two or more team members engage with the source of distraction by verbally responding while maintaining primary task activity
7	Sterile team’s work interrupted—operation flow disrupted: interruption of the current primary task of the sterile team, the operation flow is disrupted

### Statistics

To facilitate statistical analysis, the recordings were annotated with The Observer® XT 11.5 software (Noldus Information Technologies, Wageningen, The Netherlands). To assess the interobserver variability, a random sample of six recordings was scored by both observers. The findings of the two observers for these six procedures were compared and the interobserver agreement was calculated (compares events between two observations and takes the frequency and sequence into account; function incorporated in The Observer® XT 11.5 software). After satisfactory interobserver agreement was achieved, the remaining procedures were annotated by either one of the two observers (randomly allocated and analyzed in a non-chronological random order) [23, 24]. For statistical analysis, SPSS 23 statistical software was used. Intraclass correlation coefficient (ICC) was used to assess the inter-rater agreement. A two-way random effects model was used since both the procedures as well as the raters are a random sample from a larger pool of procedures and raters. We checked for consistency (i.e., raters have a similar pattern of scores). Outcomes are both average measures and single measures. Average measures provide the reliability of the score being able to separate different levels of safety, despite differences in individual scoring. Single Measures represent the reliability you would get if one rater was used. Values between 0.4 and 0.75 were considered to represent “fair to good reliability” and > 0.75 “excellent reliability” [25]. In case the kappa becomes negative (due to low variability and high agreement), the absolute agreement was described as a percentage [26]. A Pearson Chi-square test was used to compare proportions and a Mann–Whitney U test was used for continuous variables (non-normally distributed data). A *p* < .05 was considered statistically significant.

## Results

**Fig. 1:**
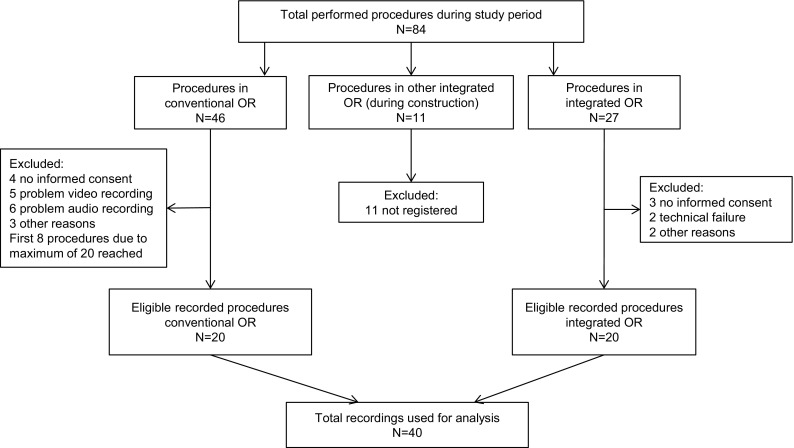
Inclusion of eligible procedures

During the study period, 84 LHs were performed of which 40 were eligible for inclusion in two studies [3]. For detailed information on the excluded procedures, see Fig. 1. All procedures were successfully completed and three minor postoperative complications were noted (Tables 3, 4).

**Table 3:** Patient and procedure characteristics of analyzed LHs performed in the Haaglanden Medical Center, The Hague, between January 2011 and April 2012

	Overall (*N* = 40)		
	Median	IQR	Min–max
Age (years)	48.2	43.9–55.2	
BMI (kg/m^2^)	24.9	22.7–27.3	
Uterine weight (g)	165	97–256	
Operating time (min)^a^	121	± 29	66–176
Procedure time (min)^b^	156	± 31	98–215
Estimated blood loss (mL)	100	50–175	
Hospital stay (days)	2.0	1.1–2.1	
Benign indication (%)	70.0%		

*IQR* inter quartile range (25th and 75th percentile), *BMI* Body Mass Index

^a^Time between first incision and last suture (skin-to-skin) (based on video observation)

^b^Time between patient entering OR and leaving OR (based on video observation)

**Table 4:** Adverse events all analyzed LHs

	Overall (*N* = 40)
Infection	1 (2.5%)^a^
Blood loss > 1L	1 (2.5%)^b^
Others	1 (2.5%)^c^
Total	3 (7.5%)

All adverse events did not require re-operation and occurred postoperatively

*LH* Laparoscopic hysterectomy

^a^Urinary tract infection

^b^Postoperative drop in hemoglobin. CT-scan showed free fluid intra-abdominally. Vital signs were stable and after a blood transfusion with two packed cells hemoglobin levels remained stable

^c^Patient suffered from sensibility loss in her right hand. The neurologist diagnosed a neurapraxia of the median nerve. Conservative management resulted in almost complete recovery

The (assisting-)surgeon answered 95% of all questions [494 out of total 520 questions (40 procedures, 13 questions per procedure)], the scrub nurse answered 89% (461 out of 520), and the anesthetist(-assistant) answered 86% of the questions (445 out of 520). Based on the questionnaire, all surgical team members were of the opinion that the study did not interfere with the procedure in 33 out of the 40 procedures (83%). In all cases, one of the two experienced gynecologists (> 100 LHs) attended the procedure. Nevertheless, the questionnaire was filled out in 58% of the cases by the assisting surgeon. As a result, reported experience of the surgeon with LH varied between ≤ 25 prior procedures (14%), 26–40 (30%), 41–100 (32%), and > 100 prior LHs in 24% of the procedures. The surgeons reported in 41% of the cases to have used the same instruments and devices > 100 times before in prior procedures. In 50% they reported to have experience with the equipment between 25 and 100 prior procedures and in 8% this was ≤ 25 procedures. Experience of the scrub nurse with MIS was in 37% of the cases between 41 and 100 and in 53% > 100 prior procedures. Despite this, experience with LH specifically was moderate; in 71% of the cases the scrub nurse had performed ≤ 25 prior LH procedures. Similarly, their experience with the equipment was moderate (in 43–47% of the cases ≤ 25 procedures).

### Surgical Safety Questionnaire

The scores per question of the individual team members are summarized in Table 5. In 15% (6 out of 40) of the procedures, potential safety concerns [i.e., answer ‘*indifferent*’ or ‘(*strongly*) *disagree*’] were reported regarding the time-out and sign-out procedure. With respect to the preparation, functioning, and ease of use of the devices in 37.5–47.5% (15–19 out of 40 procedures) a potential safety concern was reported by one or more team members. A strong disagreement to a flawless use of the devices was reported in seven procedures (17.5%). With respect to communication and collaboration in 30–35% (12–14 out of 40 procedures) concerns were reported, mostly by the surgeon.

**Table 5:** scores per question of the team members individually

Question	Surgeon				Scrub nurse				Anesthetist			
	*N*	Mean ± SD	Range	*N* ≤ 3	*N*	Mean ± SD	Range	*N* ≤ 3	*N*	Mean ± SD	Range	*N* ≤ 3
Time-out	39	4.54 ± 0.55	3–5	1	36	4.19 ± 0.67	2–5	3	37	4.08 ± 0.68	2–5	3
Sign-out	37	4.49 ± 0.51	4–5	0	31	4.16 ± 0.86	2–5	5	28	3.96 ± 0.51	2–5	2
Preparation	39	3.97 ± 1.06	1–5	11	36	4.14 ± 0.72	2–5	5	34	3.88 ± 0.81	2–5	7
Functioning	39	3.51 ± 1.21	1–5	16	36	3.83 ± 1.11	1–5	6	33	3.85 ± 0.67	2–5	6
Ease of use	39	3.82 ± 1.07	1–5	11	36	3.94 ± 0.83	2–5	5	32	3.81 ± 0.74	1–5	7
Communication	39	3.9 ± 0.75	2–5	11	35	3.86 ± 0.77	2–5	5	36	4.11 ± 0.52	3–5	3
Collaboration	39	3.92 ± 0.74	2–5	10	36	3.89 ± 0.62	2–5	5	36	4.14 ± 0.42	3–5	1
Disturbances	39	3.95 ± 0.92	2–5	7	36	3.89 ± 0.85	1–5	4	35	3.77 ± 0.81	2–5	8
Surgeon	28	3.96 ± 0.43	3–5	3	36	4.25 ± 0.55	3–5	2	35	4.14 ± 0.49	3–5	2
Scrub nurse	39	3.92 ± 0.62	2–5	7	35	4 ± 0.48	3–5	4	35	4.14 ± 0.43	3–5	1
Anesthetist	39	4.18 ± 0.51	3–5	2	36	4.19 ± 0.47	3–5	1	32	4.41 ± 0.5	4–5	0
Patient safety	39	4.21 ± 0.7	3–5	4	36	4.08 ± 0.5	2–5	1	36	4.42 ± 0.5	4–5	0
Study influence	39	4.56 ± 0.6	3–5	2	36	4.31 ± 0.47	4–5	0	36	3.97 ± 0.81	2–5	6

*N* ≤ 3: The number of questions to which a score ≤ 3 was given, which is defined as a safety concern

In general, scores given by the surgeon were in 16% (85/520) regarded as a potential safety concern. With respect to the scrub nurse and anesthesiologist this was both 9% (46/520). Overall, ‘*strongly disagree*’ was reported in 2% (9/520), of which 8 were reported on questions 3, 4, or 5 (i.e., equipment related, see Online Appendix).

In 87% (452 of 520 questions), all members of the surgical team agreed in their answers (i.e., the maximum difference between the lowest and the highest was ≤ one point on the Likert scale). In 4% (22 of 520), the absolute difference between the members of the surgical team was high (≥ 3; for example, to the same question the surgeon reports ‘*disagree*’ and the scrub nurse reports ‘*strongly agree’*). The ICC was 0.44 (average measures).

### Validation of Surgical Safety Questionnaire by video analysis

The overall observation duration of these procedures was 103 h and 45 min. Six randomly chosen observations were annotated by both observers and showed excellent agreement (Cohen’s Kappa of 0.79–0.98, all observations combined 0.85, *p* < .001). Therefore, the remaining procedures were annotated by the two observers separately (in total 36 observations by M.D.B. and 10 by S.R.C.D., respectively). The duration and effect of disturbances during procedures in which a potential safety concern was reported with regard to the functioning of devices and instruments (question 4, see Online Appendix) were compared to the procedures in which no safety concern was reported (Table 6). In the procedures after which a potential safety concern was reported, a significantly higher percentage of the duration of the procedure consisted of surgical flow disturbances [9.3 ± 6.2 vs. 2.9 ± 3.7% (mean ± SD), *p* < .001]. Similarly, in these procedures, a significantly higher mean weighted effect (i.e., the mean effect of the disturbances corrected for the duration of the disturbances) was found (score 6.1 ± 1.9 vs. 4.4 ± 2.4, *p* = .020; see Table 2 for the meaning of the scores).

**Table 6:** Duration and effect of surgical flow disturbances with regard to functioning of devices and instruments (question 4 of questionnaire) separated between procedures with or without a safety concern reported by at least one member of the surgical team (*N* = 40 procedures)

	No safety concern reported^d^			Safety concern reported^d^			
	*N*	Mean ± SD	Min–max	*N*	Mean ± SD	Min–max	*p*_e_
Percentage of procedure^a^	21	2.9 ± 3.7	0.0–15.4	19	9.3 ± 6.2	1.6–21.7	< .001
Effect (weighted)^b^	21	4.4 ± 2.4	0.0–7.0	19	6.1 ± 1.9	3.0–7.0	.020
Impact^c^	21	13.2 ± 12.0	0.0–47.1	19	56.2 ± 38.7	11.5–145.7	< .001

*SD* standard deviation

^a^Total duration of the disturbance (s) defined as percentage of the total procedure time

^b^Effect of the disturbance (based on Persoon et al. [9]) corrected by the duration of the disturbance(s)

^c^Percentage of procedure multiplied by weighted effect

^d^Reported answer by at least one surgical team member was (strongly) disagree or indifferent

^e^Mann–Whitney U test for independent samples

In the group without any reported safety concerns, there were only two procedures during which a relatively high percentage of the procedure consisted of disturbances (10.0 and 15.4%, respectively). However, the mean weighted effect of these disturbances was low (1.9 and 3.0, respectively) and therefore can be regarded as adequately managed. All tests to assess whether using the questionnaire of one or two of the team members might be applicable as well resulted in lower agreement with the video analysis (not shown).

### Newly introduced devices and/or technology

During eight procedures (20%, four procedures in the conventional OR and four in the integrated OR), a new instrument and/or device was used. During these procedures, the surgical team members reported a potential safety concern in 51% (41 out of 80 questions regarding intraoperative aspects (question 3 till 12), see Online Appendix). In contrast, the prevalence of a potential safety concern during the other procedures was 23.1% (74 out of 320, *p* < .001).

The first 20 procedures were performed in a conventional cart-based OR. The last 20 procedures were performed in a new integrated OR. No difference in potential safety concerns was reported between the two OR set-ups (28 vs. 29%, *p* = .740). Furthermore, an employee of the medical industry was present during seven procedures (four in conventional OR, three in integrated OR), during which a newly introduced device was used. Additionally, in one procedure a new device was used without an employee of the industry being present (fourth consecutive procedure in which this instrument was used). The new equipment concerned a new bipolar sealing instrument (five procedures), a new type of suture for the vaginal cuff (one procedure), and multiple new devices/instruments (three procedures).

### Experience

Limited experience of the scrub nurse with the equipment (≤ 25 procedures) resulted in significantly more potential safety concerns reported by at least one member of the surgical team (30.7 vs. 15.6%, *p* = .002). However, this did not result in a higher percentage of procedure time expended to surgical flow disturbances (7.3 ± 7.6 vs. 5.0 ± 5.2%, *p* = .423) and/or a higher effect of these disturbances (5.7 ± 1.4 vs. 4.8 ± 2.3, *p* = .275) (*N* = 30 procedures). Experience of the surgeon with the used instruments did not have a significant influence on the potential safety concerns either (25.6 vs. 23.8%, *p* = .791).

## Discussion

The Surgical Safety Questionnaire filled out directly postoperative by all members of the surgical team proved to be a valid tool to adequately estimate surgical safety in MIS. Procedures during which a relatively high percentage of the duration consisted of surgical flow disturbances and/or with a relatively high mean weighted effect of these disturbances matched with the reported potential safety concerns. Furthermore, during procedures in which a new instrument or device was used, significantly more potential safety concerns were reported by the surgical team. Therefore this could be a useful tool in the evaluation and maintenance of surgical safety during the introduction of new surgical equipment or technology.

The term patient safety is at risk to become an empty phrase by its broad interpretation. To define nuances in patient safety, the ‘systems approach’ is most commonly used [27, 28]. Based on this approach, several studies introduced frameworks covering the risk domains relevant to surgical safety and patient outcomes [6, 7, 29]. The questionnaire validated in present study covers these risk domains and thereby provides a composite outcome for surgical safety.

A study conducted by Russ et al. had similar objectives and described the Metric for Evaluating Task Execution in the Operating Room (METEOR) as an easy to use tool to allow surgical teams to self-assess their performance, in order to track surgical hazards, and to be able to evaluate safety [30]. However, their checklist is quite extensive (up to 80 items) and does not cover concerns regarding instruments and devices. Since the high dependency on technology in MIS, equipment-related disturbances are one of the well-known primary sources of disruption [3, 8, 31]. Additionally, during the introduction of a *new* technique and/or technology in the OR, disruptions are even more likely to occur [4, 7]. This hazard is also one of the main results in our study. Therefore, prior to the introduction of a new intervention in the OR, a prospective risk analysis should be performed to guarantee safe implementation (e.g., Healthcare Failure Mode and Effect Analysis) [32]. Nevertheless, in our opinion, methods currently used to monitor this implementation (i.e., evaluation after 6 and 12 months, adverse events registration, incident reporting system) fail to detect safety concerns in a timely manner. Similarly, our results rule out the widespread assumption that an employee of the medical industry being present can prevent safety hazards. Instead, the Surgical Safety Questionnaire presented in this study could be a useful tool to systematically evaluate the surgical safety after each procedure, especially in case of the introduction of a new instrument or technology.

The main strength of our study is that by using video observation we were able to assess surgical flow disturbances without influencing the course of the procedure. In that way, we obtained very reliable quantitative results to serve as *gold standard* and thereby allowing validation of our Surgical Safety Questionnaire. This is in line with other studies recognizing the additional value of detailed analysis of video registration [33, 34]. A weakness could be that scoring on a 5-point Likert scale remains prone to subjectivity. What determines the difference between agree, neither agree nor disagree, and disagreement? It was decided to place the cut-off for a potential safety concern at ‘neither agree nor disagree.’ By doing so, every time at least one of the team members for any reason had a motive to not (fully) agree on a certain question in the questionnaire, the item was marked as potential safety concern. Nevertheless, the results of our study indicate that by using this definition the potential safety concerns correlate very well with the observed surgical flow disturbances. Furthermore, in contrast to the high agreement (87%), the reported ICC (0.44) seems low. However, this discrepancy is explained by the low variability and high agreement in the reported answers. In those cases, kappa is not a reliable estimate for correlation [26]. Thirdly, the reported experience with the LH seems low. This is due to the system in The Netherlands, in which residents specializing in MIS are usually allowed to perform LH as ‘primary’ surgeon during the last year of their residency and therefore also filled out our scoring sheets. However, without exception, in these cases, the senior consultant with extensive experience in advanced gynecologic endoscopy was always member of the sterile team as well.

Over the past decades patient outcomes regarding MIS have rapidly improved. Large leaps could be made in the early days of MIS, where measures taken to improve safety were highly effective. Currently, only smaller steps can be made with a higher risk of doing harm instead of good [1, 35]. Furthermore, the OR has become increasingly complex. As Sir Cyril Chantler said: “Medicine used to be simple, ineffective and relatively safe. Now it is complex, effective and potentially dangerous” [36]. The common objective we are pursuing is to enable technology to assist the surgeon and its team in maintaining surgical safety. Similar to recent developments in the automotive industry to assist the driver on traffic safety (e.g., collision avoidance, blind spot detection, and lane departure warning systems), some promising systems are currently tested in a few hospitals in The Netherlands. For example, the Digital Operating Room Assistant continuously monitors the location, status, and (mal)functioning of devices [37, 38].

In conclusion, the results of our study demonstrate that the presented Surgical Safety Questionnaire can act as a validated tool to evaluate and maintain surgical safety during minimally invasive procedures. In daily practice, we recommend to fill out this questionnaire in case a new technique or technology is used during a procedure. By involving the complete surgical team with their individual knowledge, experience, and opinions, this will provide the opportunity to constantly evaluate new equipment and techniques. As a consequence, in an early stage, potential safety hazards will be prevented in future patients.

## Electronic supplementary material

Below is the link to the electronic supplementary material.

Supplementary material 1 (DOC 76 KB)
