# Supplementary material for: Measuring surgical safety during minimally invasive surgical procedures: a validation study
Source: Surg Endosc. 2018 Jan 19;32(7):3087–95. doi: 10.1007/s00464-018-6021-7 (PMC5988766; doi:10.1007/s00464-018-6021-7)
Supplement: Supplementary file 1 — Supplementary material 1 (DOC 76 KB) [file 464_2018_6021_MOESM1_ESM.doc]

Strongly Strongly

disagree Disagree Indifferent Agree agree

**1. The time-out procedure was followed according to protocol 🞎 🞎 🞎 🞎 🞎**

(e.g.: completeness, presence, inaccuracies)

**2. The sign-out procedure was followed according to protocol 🞎 🞎 🞎 🞎 🞎**

(e.g.: completeness, presence, inaccuracies)

**3. The preparation of devices and instruments was optimal** **🞎 🞎 🞎 🞎 🞎**

(e.g.: presence, checks passed, time-loss)

**4. The functioning of devices and instruments was optimal** **🞎 🞎 🞎 🞎 🞎**

(e.g.: problems, complexity and time-loss in case of problem)

**5. The ease of use of devices and instruments was optimal** **🞎 🞎 🞎 🞎 🞎**

(e.g.: ease of installation, intuitivity)

**6. The communication in the complete OR-team was optimal** **🞎 🞎 🞎 🞎 🞎**

(e.g.: communication failures identified, time-loss, consequences)

**7. The collaboration within the complete OR-team was optimal** **🞎 🞎 🞎 🞎 🞎**

(e.g.: possibilities for improvement)

**8. During the procedure no disturbing / distracting factors occurred 🞎 🞎 🞎 🞎 🞎**

(e.g.: unnecessary door movements, irrelevant conversations, pager / telephone)

**9. The surgeon had a professional attitude** **🞎 🞎 🞎 🞎 🞎**

(e.g.: leadership, teacher, surgical skills, communication, collaboration, possibilities for improvement)

**10. The scrub nurse had a professional attitude** **🞎 🞎 🞎 🞎 🞎**

(e.g.: preparation OR**,** communication, collaboration, active participation, takes responsibility)

**11. The anesthetist(-assistant) had a professional attitude** **🞎 🞎 🞎 🞎 🞎**

(e.g.: preparation OR**,** adequate anesthesia, communication, collaboration)

**12. The patient safety during the whole procedure was optimal 🞎 🞎 🞎 🞎 🞎**

**.**

**13. The IMPALA-study has no influence on the course of the procedure 🞎 🞎 🞎 🞎 🞎**

(e.g.: awareness presence camera / microphone, communication, atmosphere)

**Z.O.Z. Z.O.Z.**

**How much experience do you have with**:

This procedure 0-10 11-25 26-40 41-100 >100

Laparoscopy in general 0-10 11-25 26-40 41-100 >100

The used instruments 0-10 11-25 26-40 41-100 >100

The used devices 0-10 11-25 26-40 41-100 >100

**Present procedure:**

Adhesions: none few moderate extensive

Adverse events yes/no: _____________________________________________________________________________________________

________________________________________________________________________________________________________________

Additional remarks: ________________________________________________________________________________________________

________________________________________________________________________________________________________________________________________________________________________________________________________________________________

Thank you for you co-operation!
